# Stressful Life Events, Depression, and Non-Suicidal Self-Injury Among Chinese Left-Behind Children: Moderating Effects of Self-Esteem

**DOI:** 10.3389/fpsyt.2019.00244

**Published:** 2019-04-17

**Authors:** Tian Lan, Xuji Jia, Danhua Lin, Xia Liu

**Affiliations:** ^1^Faculty of Psychology, Beijing Normal University, Beijing, China; ^2^Tianjin Normal University, Tianjin, China

**Keywords:** Chinese left-behind children, stressful life events, self-esteem, left-behind type, depression, non-suicidal self-injury

## Abstract

Using cross-sectional data of the children in the Guizhou Province of China, the present study examined the association between stressful life events and self-esteem, depression, and non-suicidal self-injury (NSSI). The participants included 1,210 children recruited from four junior high schools. Self-report questionnaires concerned stressful life events, self-esteem, depression, and NSSI. Results indicated that Chinese left-behind children who faced more stressful life events were more likely to engage in depression and non-suicidal self-injury (NSSI). Moreover, self-esteem moderated the association between stressful life events and depression, and the association between stressful life events and NSSI. For the left-behind children reporting higher levels of self-esteem, the negative effect of stressful life events on depression and NSSI appeared weaker, compared to those who reported lower levels of self-esteem. In addition, the left-behind type also has a moderating effect on the relationship between stressful life events and NSSI. With the increase of stressful life events, the NSSI among the one-migrating-parent group grows more rapidly than that among the two-migrating-parents group. The findings suggest that self-esteem and left-behind type are important individual factors for Chinese left-behind children.

## Introduction

Left-behind children, a special group of children in China, has aroused widespread concern in Chinese society currently. The left-behind children refer to minors under the age of 18, and one of their parents or both their parents migrate out in search of work in big cities far away from their home, which makes their living environment lack the companionship of both their parents (1, 2). Usually, due to financial reasons, the children cannot live together with their parents in the workplace. Although the parents sometimes communicate with their children on the phone, in order to make more money, they usually go home once a year, or even several years and stay at home for about 1 week. According to the data in 2017 (3), there have been over 68 million left-behind children in China, which is a very large number that needs to be taken seriously. At present, the Chinese government attaches great importance to left-behind children. In 2013, the Chinese Ministry of Education and other five departments issued the opinions on strengthening the rural left-behind children care and education work in the stage of compulsory education. In 2016, opinions on strengthening care and protection of rural left-behind children were released by the Chinese State Council. Compared with other children, the biggest difference of left-behind children is that their parents are not around, which leads to their difficulties in daily life. For instance, many studies have indicated that compared with the children whose parents are around, left-behind children suffer more difficulties, such as stress and discrimination (4–7), and usually the negative perception of stress and discrimination will cause other emotional and behavioral problems (8, 9). Thus, in order to give left-behind children more protection and help, studies to find the relationship between stress and adaptive outcomes are very important.

### Depression and Non-Suicidal Self-Injury Among Left-Behind Children

Depression as an important variable to distinguish adolescents’ mental health has attracted much attention in recent years. According to the research of depression therapies, depression might affect 2%–8% of children and adolescents (10), which is a very large number. It was found that disadvantaged children were more vulnerable to depression than normal children. For example, research pointed out that compared with the common children, depression was significantly higher among migrant children (11). For the left-behind children, due to the lack of parental companionship, they might face more problems in daily life, which causes them to be more prone to depression. The previous studies have shown that depression is a high-emotional problem among left-behind children (12–14). Compared with other children, it has been certified that depression is more likely to occur among left-behind children (12, 15, 16). Therefore, it is of great significance to investigate what factors lead to the depression of left-behind children.

In addition to internalizing problems such as depression, left-behind children also show externalizing problems due to their special situation and identity. Previous studies have revealed that compared with the children whose parents are at home, the left-behind children often show unfavorable behaviors to their development, such as problem behavior (17, 18) and Internet game disorder (19). However, currently, additional serious behavioral problems begin to attract the attention of researchers, for example, non-suicidal self-injury (NSSI) (20).

NSSI means without suicidal intent, individuals directly and deliberately destroy their own body tissue, which occurs at an alarming rate among both clinical and community samples. This behavior is not accepted by society and is not fatal or has a low fatality rate (21–23). Usually, NSSI includes cuts, burns, scratches, and so on (24, 25). A large number of studies have shown that children and adolescents are at high risk of NSSI (26–28). According to research, 17.2% of adolescents engage in NSSI (25). Moreover, previous studies demonstrated that family structure was an important variable associated with NSSI: children from divorced families and reconstituted family had a higher incidence of NSSI than those from intact families (29, 30). As for left-behind children, the absence of parents results in them having an incomplete family structure and a disadvantaged environment, which makes them more prone to NSSI (31). But so far, few studies specifically discussed the mechanism of how NSSI appeared among left-behind children. Thus, for the harmfulness of NSSI, and for the specialty of left-behind children, it is necessary to explore the influencing factors of NSSI, so as to better help society to solve the problems faced by left-behind children.

### Stressful Life Events, Depression, and Non-Suicidal Self-Injury

Among the factors influencing depression and NSSI, stress is an important risk factor (32, 33). As a variable to quantify stress, stressful life events cover many aspects of stress, such as interpersonal relationship, academic stress, and other dimensions (34). According to previous research, stressful life events are important risk factors for both depression and NSSI. For instance, according to a longitudinal study, stressful life events were significantly associated with depressive symptoms (35). Besides, it revealed that stressful life events corresponded with higher levels of depressive cognitions (36). As for NSSI, a study found that stressful life events were associated with risk for NSSI in adolescents (37). In addition, it revealed that there was a significant positive relationship between stressful life events and NSSI among middle school students (38). It also showed that, for teenagers, stressful life events had a significant prediction on junior high school students’ NSSI (39).

As for the left-behind children group, the previous studies have revealed that compared with the children whose parents stay at home, the left-behind children get more stress (40), which may have a more negative impact on their psychological adaptation (4). In addition, according to the results found in the left-behind children group, stressful life events significantly predicted left-behind children’s internalizing and externalizing problem behavior. For example, it has found that stressful life events can significantly predict internalizing problems such as loneliness (9). Meanwhile, stress is also the main predictive variable of externalizing problems such as anti-social problem behaviors and online behaviors (41). However, there are still few studies on the relationship between stressful life events and depression and NSSI in left-behind children. Thus, for left-behind children, whether stressful life events will lead to an increase in depression and NSSI will be discussed in the present research.

### The Moderating Function of Self-Esteem

Although negative factors will cause more depression and NSSI among children, there are still individual differences; whether the child has protective factors matters a lot (42, 43). Children’s self-cognition plays an important moderate role; for example, self-esteem is thought to have a significant effect in promoting children’s positive adaptation (44, 45). Self-esteem is an emotional evaluation of the individual himself (46). It means that people who have high self-esteem usually have a positive evaluation of themselves. They are more confident in life and will face difficulties bravely. While for the people who have low self-esteem, they have a negative view of themselves, lack the confidence for future, and when facing difficulties, they do not have enough courage (47–49). Self-esteem has been discussed in many children groups, such as migrant children (50) and AIDS-affected children in rural areas (51). The results indicated that self-esteem was one of the important aspects to moderate the children’s adaptation problem. As for research about the left-behind children, the previous studies indicated that self-esteem could buffer the relationship between social support and depression of the left-behind children (52). But there are few studies of self-esteem on the moderating function between the left-behind children’s stressful life events and depression and NSSI. Considering the importance of self-esteem to the psychological adaptation of children, the question arises whether self-esteem affects the relationship between stressful life events and depression, as well as between stressful life events and NSSI. To answer this question, the relationship between the three variables will be discussed in the present research.

### The Moderating Function of Gender and Left-Behind Type

In addition, the existing studies have found that the gender factor is one of the important adjustment variables that affect the left-behind children’s emotion and behavior (53, 54). For instance, it found that compared with left-behind girls, left-behind boys showed more externalizing problem behavior (55, 56). While on the contrary, when facing stressful events, left-behind girls always showed more negative emotional response and internalizing problem behavior (57, 58). Hence, when meeting the same negative events, left-behind boys and left-behind girls may show a different response, which means gender may have a moderating function on the relationship between stressful life events and depression, as well as between stressful life events and NSSI.

In addition, recently, more and more studies are focusing on the unique variables of left-behind children—left-behind type (57, 59, 60). According to the case of parents’ out, the left-behind children can be divided into two groups—one-migrating-parent group and two-migrating-parents group. Previous studies had explored the differences between the two groups, and results indicated that depression was significantly different between the one-migrating-parent group and the two-migrating-parents group (61). Meanwhile, it is found that when the mother is out for work, the incidence of NSSI is high (62). The present results mean that different left-behind type may have different functions on left-behind children’s depression and NSSI. Therefore, it is necessary to explore the moderating function of left-behind type between left-behind children’s stressful life events and depression, as well as between stressful life events and NSSI.

### The Present Study

To sum up, the present study has two main purposes. First, we will explore the relationship between stressful life events and depression, as well as the relationship between stressful life events and NSSI among left-behind children. Second, the study will investigate whether self-esteem, gender, and left-behind type play a moderate role in the relationship.

According to the two purposes above, we anticipate that stressful life events and depression have significant correlations. Also, there is a significant correlation between stressful life events and NSSI. We hypothesize that self-esteem significantly moderates the relation of stressful life events and depression, as well as the relation of stressful life events and NSSI, which means that children who have high self-esteem will have less depression and NSSI. Besides, we anticipate that gender and left-behind type will also have a moderating function between the relationship of stressful life events and depression, as well as the relationship between stressful life events and NSSI. Through this study, we hope to find critical influencing factors of the left-behind children’s depression and NSSI.

## Method

### Participants

A total of 1,282 left-behind children were invited, and 1,210 children were eventually selected for the study (591 boys, 596 girls, and 23 lost). The response rate was 94.4%. The participants were recruited from four junior high schools in a rural county of Guizhou Province, which is an area of China where many people have migrated for work so that a lot of children are left at home. The mean age of boys was 13.63 (*SD* = 1.06; range = 11–19 years) and that of girls was 13.45 (*SD* = 1.13; range = 11–18 years). No child had obvious physical or developmental disabilities.

Specifically, among the left-behind children, the number of one-migrating-parent children was 363, and their mean age was 13.59 years (*SD* = 1.05; range = 12–17 years). The number of two-migrating-parents’ children was 304, and their mean age was 13.56 years (*SD* = 1.06; range = 11–19 years). As for the frequency that parents came back home, 10.5% of the parents came back home once a month or more than once a month, 10.5% of the parents came back home once every 2 or 3 months, 19.6% of the parents came back home every half a year, and 50.4% of the parents came back home once a year or once every several years. During the period that the parents did not come home, 0.9% of the parents never contacted their children, 24.7% of parents contacted their children once a month or once every several months, and 65.1% of parents contacted their children more than once a month. A total of 94.1% of the fathers and 94.4% of the mothers had an education level of junior high school or lower in the group with one migrant parent, and in the two-migrating-parents group, 91.5% of the fathers and 95.3% of the mothers had an education level of junior high school or lower. The number of no-migrating-parent children was 543, and their mean age was 13.49 years (*SD* = 1.16; range = 11–18). A total of 87.9% of the fathers and 93.6% of the mothers had an education level of junior high school or lower in the group with no migrant parents.

### Procedures

Participants were recruited *via* four schools in Guizhou Province, which has a high percentage of left-behind children. Before the investigation, we first contacted the school principal. For those schools that were willing to participate in the survey, we sent the consent 2 months in advance to parents. The parents were required to complete the consent. If neither parent is at home, it would be completed by the family members who take care of the children. After the consent was collected, we distributed the questionnaire to the children whose parents agreed to participate in the survey.

The students were divided into groups to complete the questionnaires after the school’s permission of performing the study. Each group was given instructions by experimenters. It was certain that the participants had the ability to understand the items and the procedure on the questionnaires. For each question, there were no right or wrong answers. Besides, the students were told that all the information they answered in the study would be kept secret. All the students completed the questionnaire independently, and the experimenters would take back the questionnaire separately after completing the questionnaire to avoid information leakage. After completing the surveys, participants received a prize (T-shirt, pens, and notebooks).

In order to ensure the scientific nature of the study, all measurements were carried out by psychologists and psychology students at Beijing Normal University in China. We complied with the latest version of the Declaration of Helsinki. The study protocol was approved by the Institutional Review Board at Beijing Normal University in China.

### Measures

*Stressful life events.* The stressful life events of the left-behind children were assessed by a scale specially designed for Chinese children and adolescents (63), which has been widely used among Chinese left-behind children (41, 64, 65). Twenty-seven questions were measured in the scale including six dimensions—interpersonal relationship, academic stress, being punished, loss, health adaptation, and other else. All the questions ranged from 1 (never happened) to 6 (extremely serious influence). Higher average scores indicate higher levels of stressful life events. In our present research sample, the Cronbach’s alpha coefficient was found to be 0.89.

*Self-esteem.* The children’s self-esteem was measured by the scale designed by Rosenberg (66), which has been widely used among Chinese children and adolescents (67–69), including the left-behind children (41, 70). The scale had 10 items, such as “I feel I have many good qualities.” All the items ranged from 1 (not conform at all) to 5 (very conform). The average scores were calculated, with higher scores indicating higher levels of self-esteem. In our present research sample, the Cronbach’s alpha coefficient was found to be 0.74.

*Depression.* The children’s depression was assessed by the Center for Epidemiologic Studies Depression Scale for Children (71), which has been successfully applied to children and adolescents in China (72, 73). The scale included 20 items, such as “I don’t think I can concentrate on my work.” All the questions ranged from 1 (never) to 4 (always). The results were analyzed by way of means, which indicated that the child who had a higher grade felt more depressed. In our present research sample, the Cronbach’s alpha coefficient was found to be 0.84.

*NSSI.* The children’s NSSI was measured by a shortened and modified version of the Deliberate Self-Harm Inventory (DSHI), constructed and validated by Gratz (74) and adapted to adolescents by Lundh et al. (75), which has been proven to have good reliability and validity (76). Nine items were measured in the scale, such as “Pierce my skin with sharp objects.” The scale ranged from 1 (never) to 5 (five or more than five times). The average scores were calculated; higher scores indicated a higher level of NSSI. In our present research sample, the Cronbach’s alpha coefficient was found to be 0.85.

*Socioeconomic status (SES).* SES was measured by the MacArthur Scale (77), which has been successfully used among Chinese adolescents (78). Presenting a diagram of a “social ladder” with 10 rungs, the children were asked to choose the rung. The higher rung they choose, indicating they believed they had a higher level of SES.

### Analytic Plan

Descriptive statistics and bivariate correlations among study variables were computed. Also, a series of regression analyses reported the independent and interactive associations linking stressful life events, moderator variables (self-esteem, gender, and left-behind type), and outcome variables (depression and NSSI). In the first step, control variables (age, left-behind time, socioeconomic status, and frequency of contact) were entered. Then, predictor variables (stressful life events) and moderator variables (self-esteem, gender, and left-behind type) were entered. Finally, the interaction terms between stressful life events and moderator variables (self-esteem, gender, and left-behind type) were entered in the third step.

For significant interactions, the interaction utility (79) was used to calculate the simple intercepts and simple slopes according to standard procedures (80, 81). The following analyses produced intercepts and slopes that represent the relations between the predictor variables (stressful life events) and outcome variables (depression and NSSI) at the higher (+1 standard) and lower (−1 standard) levels of the moderator variable (self-esteem, gender, and left-behind type). Predictor variables were centered for all regression analyses.

## Result

### Preliminary Analyses

First, we examined the prevalence of depression and NSSI among the participants. For depression, the four frequencies of each item were assigned a score of 0–3, with a total score of 60. A score of less than 16 indicated no depressive symptoms, and a score greater than or equal to 16 indicated that the participant had certain depressive symptoms (52, 82). According to the standard, 65.0% of the left-behind children had depressive symptoms, and 59.4% of the non-left-behind children had depressive symptoms. For NSSI, 33.7% of the left-behind children reported having engaged in NSSI at least once, while for the non-left-behind children, 33.9% reported having engaged in NSSI at least once.

**Table 1** shows the means and standard deviations of key variables by gender and left-behind type. The results showed that the NSSI among the one-migrating-parent group was significantly higher than that among the two-migrating-parents group (*p* < 0.01).

**Table 1:** The means and standard deviations of key variables by gender and left-behind type.

	Gender			Left-behind type		
	Boys	Girls	*t*	Two-migrating-parents group	One-migrating-parent group	*t*
Stressful life events	2.17 (0.63)	2.17 (0.61)	0	2.16 (0.62)	2.20 (0.62)	0.74
Self-esteem	3.49 (0.64)	3.38 (0.63)	2.07	3.43 (0.63)	3.42 (0.64)	−0.19
Depression	1.92 (0.45)	2.09 (0.49)	−4.31	2.00 (0.47)	2.04 (0.48)	1.06
NSSI	1.13 (0.41)	1.14 (0.27)	−0.22	1.12 (0.27)	1.16 (0.42)	1.57**

**p < 0.01. NSSI, non-suicidal self-injury.

One-way ANOVA was performed on the differences among one-migrating-parent children, two-migrating-parents children, and no-migrating-parent children in the main study variables. The results are shown in **Table 2**. The three groups were significantly different on stressful life events (*F* = 3.89, *p* < 0.05). Further analysis showed that stressful life events among the one-migrating-parent group were significantly higher than that among the no-migrating-parent group (*p* < 0.05). As for depression, the three groups were marginally different (*F* = 2.84, *p* = 0.06). Further analysis showed that depression among the one-migrating-parent group was marginally higher than that of the no-migrating-parent group (*p* = 0.06). Besides, the three groups were significantly different on SES (*F* = 3.06, *p* < 0.05). The results showed that socioeconomic status among the two-migrating-parents group was significantly higher than that of the no-migrating-parent group (*p* < 0.05), but no significant differences were found on self-esteem and NSSI.

**Table 2:** The means and standard deviations of key variables.

	Stressful life events M (*SD*)	Self-esteem M (*SD*)	Depression M (*SD*)	NSSI M (*SD*)	SES M (*SD*)
One-migrating-parent children	2.20 (0.62)	3.42 (0.64)	2.04 (0.48)	1.16 (0.42)	4.21 (1.46)
Two-migrating-parents children	2.16 (0.62)	3.43 (0.63)	2.00 (0.47)	1.12 (0.27)	4.36 (1.60)
No-migrating-parent children	2.07 (0.63)	3.47 (0.66)	1.96 (0.47)	1.14 (0.33)	4.10 (1.53)
*F*	3.89*	0.76	2.84^†^	1.40	3.06*

*p < 0.05 and ^†^p < 0.1. SES, socioeconomic status.

The correlations among left-behind time, frequency of visits, frequency of contact, stressful life events, self-esteem, depression, and NSSI of left-behind children can be found in **Table 3**. The information showed that left-behind time significantly positively correlated with the frequency of visits. There was a significant and positive correlation between frequency of contact and self-esteem. However, self-esteem was negatively correlated with stressful life events, depression, and NSSI, respectively. Stressful life events were significantly positively correlated with children’s depression and NSSI, while it was significantly negatively correlated with self-esteem. Besides, self-esteem was significantly negatively correlated with depression and NSSI. Finally, there was also a significant positive correlation between depression and NSSI.

**Table 3:** The correlations among left-behind time, frequency of visits, frequency of contact, stressful life events, self-esteem, depression, and non-suicidal self-injury (NSSI).

	1	2	3	4	5	6
1. Left-behind time	—					
2. Frequency of visits	0.38**	—				
3. Frequency of contact	−0.06	−0.07	—			
4. Stressful life events	0.02	0	−0.18**	—		
5. Self-esteem	0.04	0	0.12**	−0.29**	—	
6. Depression	0.05	0.04	−0.14**	0.54**	−0.52**	—
7. NSSI	−0.04	0.02	−0.14**	0.29**	−0.24**	0.34**

**p < 0.01.

### Predicting Depression

As for the regression analyses among left-behind children, as shown in **Table 4**, after commanding the effects of control variables, stressful life events were positively and significantly associated with depression (*p* < 0.001), while self-esteem was negatively and significantly associated with depression (*p* < 0.001). Also, gender had a significant impact on depression (*p* < 0.001). In addition, among the three interaction terms (stressful life events × self-esteem, stressful life events × gender, and stressful life events × left-behind type), only one interaction term (stressful life events × self-esteem) was significant, which indicated that self-esteem moderated the relationship between stressful life events and depression. The full set of predictors explained 46% of the variance in the children’s depression. As for the no-migrating-parent group, regression analyses showed that there was no significant association between stressful life events and depression. Besides, neither self-esteem nor gender significantly moderated the relationship between stressful life events and depression. Therefore, a simple slope test was mainly conducted in the left-behind children group.

**Table 4:** Summary of the hierarchical multiple regression analyses.

	Depression			NSSI		
	*B*	*SE B*	β	*B*	*SE B*	β
Block 1						
Age	0.03	0.02	0.06	0	0.01	−0.01
Socioeconomic status	−0.02	0.02	−0.06	0.03	0.01	0.12*
Left-behind time	0.02	0.01	0.06	−0.01	0.01	−0.02
Frequency of contact	−0.07	0.03	−0.10*	−0.03	0.02	−0.07
*R* ^2^	0.02			0.01		
Block 2						
Stressful life events	0.32	0.03	0.39***	0.13	0.02	0.24***
Self-esteem	−0.31	0.03	−0.42***	−0.10	0.02	−0.20***
Gender	0.11	0.04	0.12**	0.02	0.03	0.03
Left-behind type	0.04	0.04	0.04	0.04	0.03	0.06
*R* ^2^	0.43			0.12		
Block 3						
Stressful life events × Self-esteem	−0.12	0.04	−0.11**	−0.09	0.03	−0.13**
Stressful life events × Gender	0.02	0.06	0.01	−0.07	0.05	−0.07
Stressful life events × Left-behind type	0.03	0.06	0.02	0.13	0.05	0.12*
*R* ^2^	0.01			0.03		

*p < 0.05; **p < 0.01; and ***p < 0.001.

**Figure 1:**
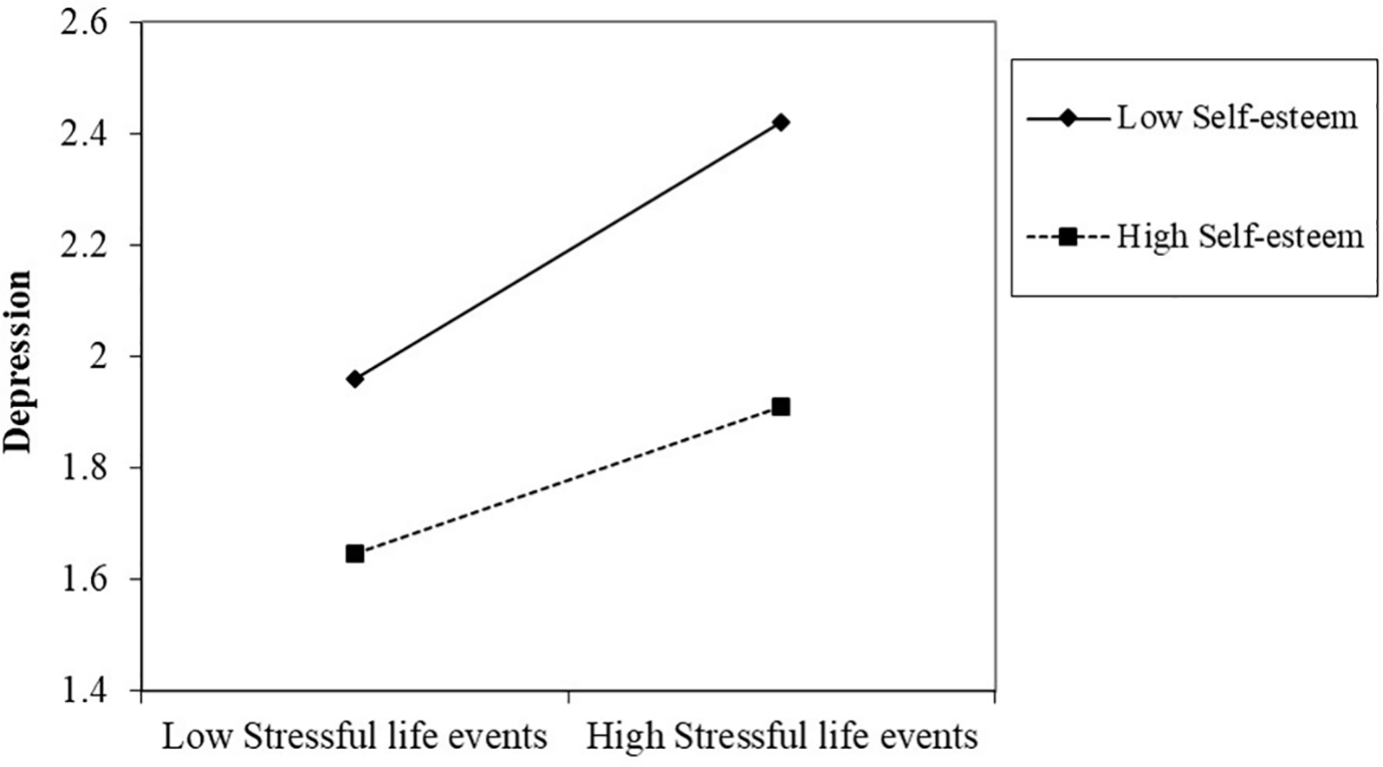
Two-way interaction of stressful life events and self-esteem on the depression of left-behind children. Low designates −1 *SD* on the scale; high designates +1 *SD* on the scale.

Simple slope analysis showed that among the left-behind children who held low levels of self-esteem, there was a significant positive correlation between stressful life events and depression (β = 0.39, *t* = 8.65, *p* < 0.001). While among the children who held high self-esteem, there was a mild relationship between stressful life events and depression (β = 0.22, *t* = 5.17, *p* < 0.001). As is shown in **Figure 1**, it is suggested that an increase in stressful life events among the children who had low self-esteem exhibited a rapid increase in depression, whereas it was milder among children with higher self-esteem.

### Predicting Non-Suicidal Self-Injury

After commanding the effects of control variables, it can be found that stressful life events had a significant and positive impact on NSSI (*p* < 0.001). On the contrary, self-esteem was significantly negatively associated with NSSI (*p* < 0.001). In addition, among all the interaction terms (stressful life events × self-esteem, stressful life events × gender, and stressful life events × left-behind type), two interaction terms (stressful life events × self-esteem and stressful life events × left-behind type) were significant, meaning that self-esteem and left-behind type moderated the relationship between stressful life events and NSSI. The full set of predictors explained 16% of the variance in the children’s NSSI. As for the no-migrating-parent group, regression analyses showed that there was no significant association between stressful life events and NSSI. Besides, neither self-esteem nor gender significantly moderated the relationship between stressful life events and NSSI. Therefore, a simple slope test was mainly conducted in the left-behind children group.

**Figure 2:**
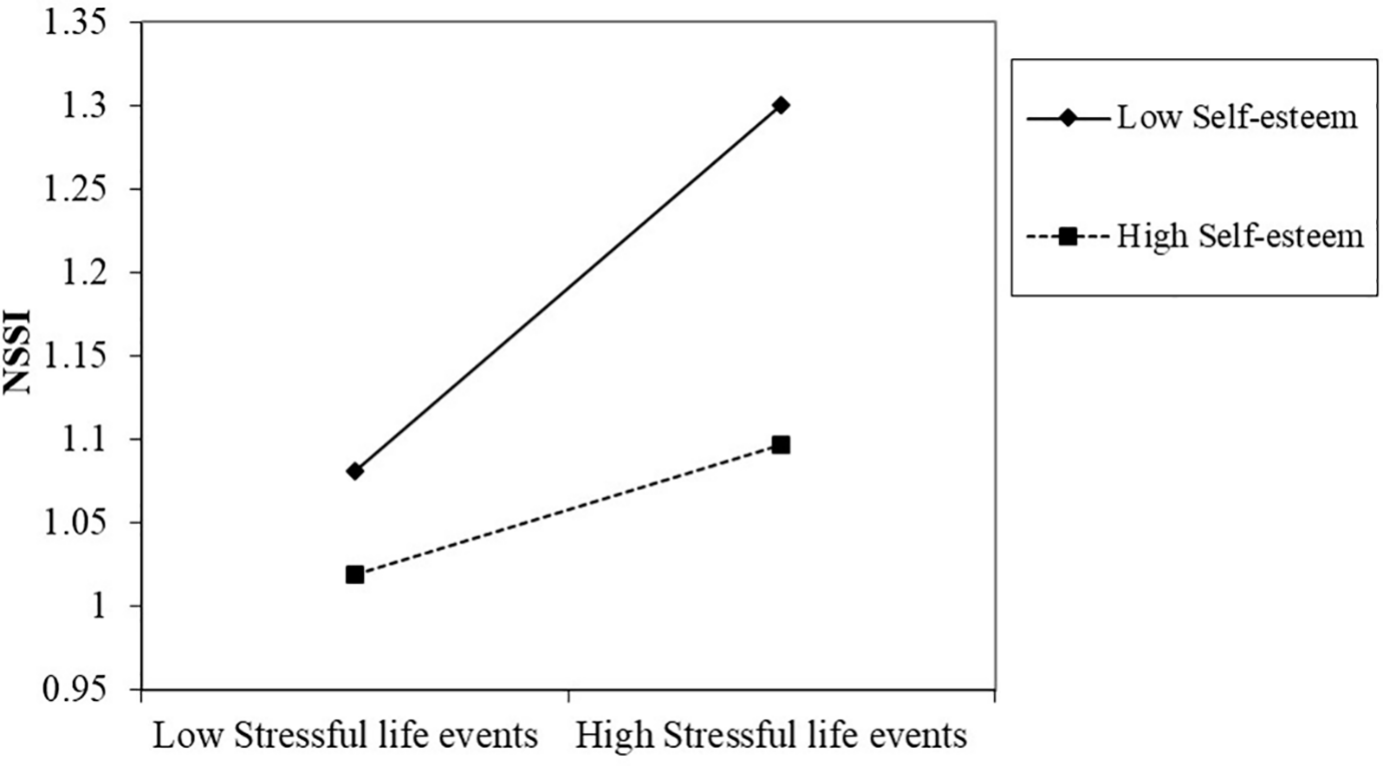
Two-way interaction of stressful life events and self-esteem on the non-suicidal self-injury (NSSI) of left-behind children. Low designates −1 *SD* on the scale; high designates +1 *SD* on the scale.

**Figure 3:**
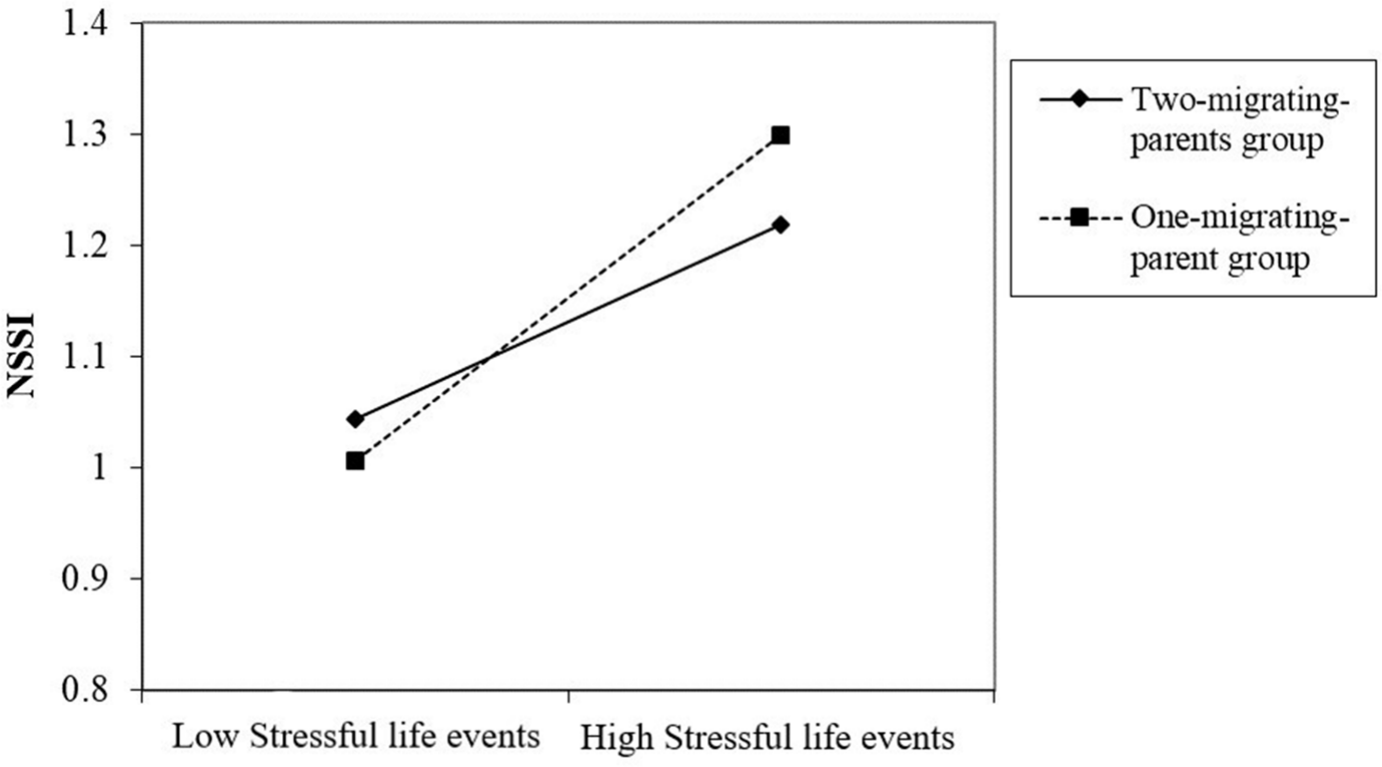
Two-way interaction of stressful life events and left-behind type on the NSSI of left-behind children. Low designates −1 *SD* on the scale; high designates +1 *SD* on the scale.

In **Figure 2**, a simple slope analysis showed that the association between stressful life events and NSSI was stronger in the low self-esteem group (β = 0.18, *t* = 3.24, *p* < 0.05) compared to the high self-esteem group (β = 0.06, *t* = 1.37, *p* > 0.05). It indicated that with the increase of stressful life events, the NSSI of the low self-esteem left-behind children showed a significant upward trend, which was mild among high self-esteem left-behind children. In addition, the simple slope analysis also revealed that the association between stressful life events and NSSI was stronger in the one-migrating-parent group (β = 0.24, *t* = 3.28, *p* < 0.05), compared with the two-migrating-parents group (β = 0.14, *t* = 3.85, *p* < 0.01), as is shown in **Figure 3**. This finding suggested that with the increase of stressful life events, the rise of NSSI in the one-migrating-parent group was rapid, whereas it was milder among the two-migrating-parents group.

## Discussion

In recent years, there has been more and more research devoted to depression and NSSI among Chinese left-behind children (14, 31, 83). The present study is the first study to discuss whether self-esteem moderates the relationship between stressful life events and depression, as well as between stressful life events and NSSI among left-behind children in China. Three essential results were found in the current study. First, there was a significant correlation between stressful life events and depression. The same relation was also found between stressful life events and NSSI. Second, self-esteem significantly moderated the relationship between stressful life events and depression, as well as between stressful life events and NSSI. For the children who have high self-esteem, with the increase of stressful life events, the level of depression and NSSI rose mildly. But for the children whose self-esteem is low, with the increase of stressful life events, the depression and NSSI showed a significantly increasing trend. Third, the left-behind type significantly moderated the relationship between stressful life events and NSSI. With the augment of stressful life events, the increase of the NSSI among the one-migrating-parent group was more rapid than that among the two-migrating-parents group.

The results showed that stressful life events among the one-migrating-parent group were significantly higher than that of the no-migrating-parent group. Besides, we found that the frequency of contact was significantly correlated with left-behind children’s stressful life events, self-esteem, depression, and NSSI. These results suggested that parents who migrate out for work should keep in touch with their children frequently, which may help them to grow up better. The study also revealed that stressful life events were positively related to left-behind children’s depression and NSSI. These findings were similar to the previous findings (20, 23, 35, 37, 84, 85). Moreover, there were paralleled findings, especially for left-behind children. For example, it was found that stressful life events had a significant correlation with depression according to research for left-behind children (86). Also, previous studies found that stressful life events affected the suicidal ideation of the left-behind children (65), which was highly correlated with depression. Our research verified and supplemented the above results. These results suggested that we should pay more attention to the negative experience of left-behind children’s lives in order to decrease the level of depression and NSSI.

Consistent with our hypothesis, self-esteem had a significant main effect on depression and NSSI. That means the higher self-esteem the children have, the less depression and NSSI they show. The results were certified in the existing studies (87–90). Low self-esteem has been considered as a risk factor for suicide, depression, and NSSI (88, 91, 92). What is more important is that due to a lack of self-regard, individuals with low self-esteem may find it easier to engage in NSSI (93). Also, self-esteem can be seen as an internalizing individual factor, which means individuals who have high self-esteem may tend to face up to the difficulties and to succeed while the low self-esteem ones were more likely to lose confidence and are prone to fail (48). Hence, for the left-behind children who have high self-esteem, they tend to use a more positive attitude toward life, while for the children with low self-esteem, they are prone to be more negative and lose self-regard, which may finally turn into depression and NSSI.

Another aim of the current study is to test the moderating function of self-esteem on the relationship between stressful life events and depression, as well as between stressful life events and NSSI. The results of the study supported our hypothesis. Specifically, we found that for children reporting higher levels of self-esteem, the increase of stressful life events will lead to a mild rise of depression and NSSI. However, for the children whose self-esteem was low, with the increase of stressful life events, depression and NSSI showed a significantly increasing trend. The result supported the view that self-esteem is a crucial individual difference variable that influences stressful life events (94). According to the vulnerability model, low-self-esteem individuals are prone to social avoidance, which may impede social support (88, 95). In China, when children face difficulties, they are more likely to seek their parents’ help in the first place. However, for the left-behind children, because of the absence of their parents, when facing stressful life events, they may need to seek other social support. While for the low self-esteem children, they avoid others’ help, which may finally cause depression and NSSI. On the contrary, children with high self-esteem will ask others’ support, which will help them to deal with stressful life events more successfully and thus compared with the children who have low self-esteem, they will accumulate less negative feelings, and the decrease of the negative feelings will keep the children away from depression and NSSI. In addition to the moderating fact of self-esteem, the left-behind type also plays an important role. The results showed that compared with the two-migrating-parents group, the children of the one-migrating-parent group seemed to be more sensitive to stressful life events. With the increase of stress, there are more NSSI behaviors among the one-migrating-parent group. The reason may be that compared with the one-migrating-parent group whose support comes from only the mother or father, most two-migrating-parents children live with their grandparents and are accompanied by more relatives. In addition, our study also found that two-migrating-parents families have higher social and economic status, and the parents usually pay more living expenses to their children and guardians, which may also reduce children’s response to stressful life events. However, it still needs further exploration.

Several limitations must be discussed in the present study. First, because the present study was conducted from the left-behind children in Guizhou Province of China, the generalization and interpretation of the present research results need to pay attention to the context. Second, variables were measured using self-report questionnaires, which may cause shared method variance and shared source. Third, the sample was cross-sectional; thus, this relationship we found in the present study cannot be thought of as causation. In spite of the limitations, the present study advances one of the first research efforts to understand the relationship between stressful life events and depression, as well as between stressful life events and NSSI among the left-behind children in China. In addition, the study showed that when discussing the link between stressful life events and depression, or exploring the relationship between stressful life events and NSSI, it is necessary to consider the children’s self-esteem and left-behind type. Further research should aim to expand the number of participants and collect samples all over the country. Also, a longitudinal design on how the relationship between stressful life events, self-esteem, left-behind type, depression, and NSSI evolve over time should be examined. In addition, the inclusion of multiple reporters should be considered in the future when assessing children’s depression and NSSI.

## Conclusion

Our present study had three main findings. First, stressful life events had a significant effect on left-behind children’s depression and NSSI. Second, self-esteem had a significant moderating function on the relationship between stressful life events and depression, as well as between stressful life events and NSSI. Third, we found that compared with the increase of stressful life events, the rise of NSSI in the one-migrating-parent children was more rapid.

## Ethics Statement

The present study was carried out in accordance with the recommendations of the Research Ethics Committee of Beijing Normal University with written informed consent from all subjects. The protocol was approved by the Research Ethics Committee of Beijing Normal University. All subjects gave written informed consent in accordance with the Declaration of Helsinki.

## Author Contributions

XL conceived and designed the study and supervised the collection of data. TL, XL, and XJ analyzed and interpreted the data, and produced the drafting of the manuscripts. DL supervised all steps in the study.

## Funding

The child development database establishment under rural-to-urban migration context and the establishment of positive youth development system, Supported by the National Social Science Foundation of China (15ZDB138).

## Conflict of Interest Statement

The authors declare that the research was conducted in the absence of any commercial or financial relationships that could be construed as a potential conflict of interest
