# Corrigendum: Stressful Life Events, Depression, and Non-Suicidal Self-Injury Among Chinese Left-Behind Children: Moderating Effects of Self-Esteem

**DOI:** 10.3389/fpsyt.2019.00685

**Published:** 2019-09-12

**Authors:** Tian Lan, Xuji Jia, Danhua Lin, Xia Liu

**Affiliations:** ^1^Faculty of Psychology, Beijing Normal University, Beijing, China; ^2^Tianjin Normal University, Tianjin, China

**Keywords:** Chinese left-behind children, stressful life events, self-esteem, left-behind type, depression, non-suicidal self-injury

In the published article, there was an error in affiliation 1. Instead of “Beijing Normal University, Beijing, China,” it should be “Faculty of Psychology, Beijing Normal University, Beijing, China.”

The authors apologize for this error and state that this does not change the scientific conclusions of the article in any way. The original article has been updated.

